# Developing and Demonstrating a Lab Method for Quantifying Hair Exposure to Environmental Tobacco Smoke with a Forensic Perspective

**DOI:** 10.1021/acs.jchemed.5c00479

**Published:** 2025-12-25

**Authors:** Tanique Z. Jones, Christopher J. Trejo, Somayeh Mohammadi, Hamidreza Sharifan

**Affiliations:** † Department of Chemistry and Biochemistry, 12337 University of Texas at El Paso, El Paso, Texas 79968, United States; ‡ Forensic Science Program, 12337 University of Texas at El Paso, El Paso, Texas 79968, United States; § Environmental Science and Engineering Program, 12337 University of Texas at El Paso, El Paso, Texas 79968, United States

**Keywords:** tobacco, smoke, hair, UV−visible, FTIR, exposure

## Abstract

Forensic science and chemistry curricula often lack hands-on experimental designs that effectively simulate the impact of environmental tobacco smoke (ETS) or other common elements found at crime scenes, such as marijuana, on trace forensic evidence. Hair, a critical form of trace evidence, offers unique advantages for assessing long-term exposure to environmental pollutants, including ETS. This study presents a novel, noninvasive forensic laboratory module designed to evaluate ETS exposure on various human hair types (untreated, dyed, and bleached). The experimental procedure involved controlled cigarette smoke exposure, followed by analysis using UV–visible spectroscopy, FTIR spectroscopy, and zeta potential measurements. Thirteen students participated in the three-week lab module (three sessions per week). Pre- and postlab assessments were conducted to evaluate learning outcomes. The prelab assessment focused on baseline knowledge of forensic hair analysis, as well as student expectations and confidence. The postlab assessment evaluated knowledge gained, technical insights, application of techniques, self-reflection, conceptual understanding, and practical skill development. This design helped students comprehend the effects of chemical treatments that significantly influence hair’s capacity to adsorb ETS residues by altering its physical and chemical properties. Integration of this experiment into the forensic chemistry curriculum led to measurable gains in student understanding, technical competency, and appreciation for real-world forensic applications. This method offers a valuable teaching and investigative tool for assessing individual ETS exposure in forensic contexts.

## Graphical abstract

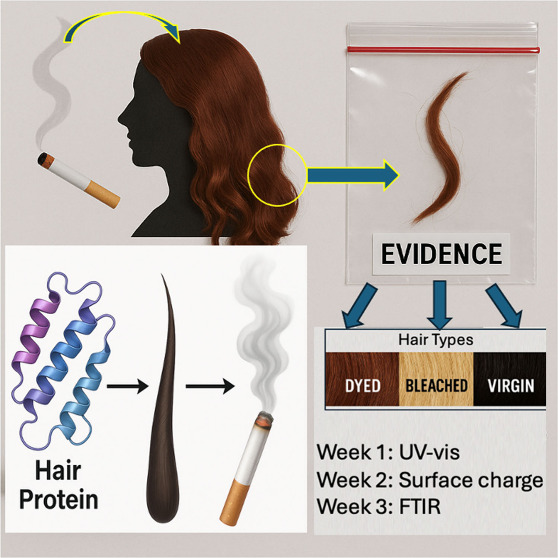

## Introduction

In forensic chemistry education, a reliable noninvasive lab module to assess cannabis and environmental tobacco smoke (ETS) exposure is critical as many crime scenes are associated with smoke and drug consumption.^1,2^ Traditional lab methods, such as blood and urine analysis, are costly, often unfeasible in many institutes and require lengthy ethical approval processes, moreover, they provide only a snapshot of recent exposure and may not reflect long-term accumulation.^3,4^ Hair analysis, on the other hand, can provide a comprehensive overview of an individual’s exposure over weeks to months, making it an invaluable tool in forensic chemistry education.^5–7^ This study aims to develop a robust lab module that enables students to effectively understand hair exposure to ETS, with a specific focus on the differential retention of smoke residues in untreated, dyed, and bleached hair. We developed an experimental setup that can be further commercialized and utilized in educational settings for forensic and environmental studies. Through teaching forensic chemistry over four years, the lead author identified the need of a lab module in forensic chemistry education to be affordable, reliable, and noninvasive for students to assess environmental exposures that play a role in legal and investigative contexts.^8,9^

ETS, commonly referred to as secondhand smoke, poses significant health risks and remains a pervasive issue in public health.^10–12^ The detrimental effects of ETS exposure, including respiratory illnesses, cardiovascular diseases, and various cancers, are well-documented.^13^ However, accurately quantifying an individual’s exposure to ETS, particularly in forensic contexts, presents unique challenges. Previous research highlights that ETS residues, specifically nicotine and its metabolites, serve as significant forensic markers in cases involving exposure assessments or custody disputes due to their persistence and detectability in biological matrices such as hair.^14,15^ Hair, as a biological matrix, offers a distinctive advantage for assessing long-term exposure to environmental pollutants,^16^ including tobacco smoke, narcotics, and marijuana.^17^ This is due to its ability to incorporate and retain various substances over extended periods, providing a historical record of exposure.^18,19^ Unlike blood or urine, which reflect recent exposure, hair analysis provides a longer window of detection, making it possible to assess chronic exposure to ETS.^20^ In addition, using the hair supply for the forensic teaching lab is low-risk and cost-effective. Further, this technique is particularly useful in cases where long-term exposure needs to be demonstrated. Hair collection is a noninvasive procedure, easily performed without causing discomfort or distress to individuals.^21,22^ This is especially advantageous in forensic contexts where minimal invasiveness is preferred. Hair has the unique ability to incorporate and retain nicotine and other smoke residues over time.^23,24^ By examining untreated, dyed, and bleached hair, this lab module can empower students to differentiate hair treatments role in the adsorption of ETS components, enhancing the accuracy of exposure assessments.^25,26^ This Lab module is flexible and integrable to improve understanding of the impact of cosmetic treatments on the absorption of environmental toxins. Through this experiment, instructors can design various forensic scenarios, including criminal investigations, workplace exposure assessments, and custody disputes where ETS exposure might be a relevant factor.^27^ It provides forensic experts with a robust tool to support their findings with scientifically validated data.^28^ This innovative approach enhances the forensic toolkit, allowing for more accurate and comprehensive exposure assessments.

## Pilot Implementation and Student Involvement

This forensic lab module was not initially part of a formal course curriculum but was practiced and evaluated with 13 volunteer undergraduate students enrolled in CHEM 4376 – Introduction to Research, FORS 3370 – Forensic Science I, and RSRC 4033 – Undergraduate Research at the University of Texas at El Paso (UTEP). Students had previously completed introductory courses in Chemistry (General Chemistry I and II). These students participated in the complete execution of the experimental procedures as part of a pilot study to evaluate the module’s effectiveness and practicality prior to its integration into the forensic chemistry curriculum. All laboratory work, including hair sample preparation and cigarette smoke exposure conducted under a fume hood in the Forensic Science Laboratory located in Lab 303 of the Physical Science Building within the Department of Chemistry and Biochemistry at UTEP. The student’s handout is provided in Supporting Information (SI), S1, showing 5 weeks of lab experiment instruction.

## Learning Goal

The primary learning goals of this laboratory module are to equip students with an understanding of forensic hair analysis as a tool for assessing environmental smoke exposure. Through hands-on experimentation, students learn technical proficiency in UV–visible spectroscopy, Fourier Transform Infrared Spectroscopy (FTIR), and zeta potential analysis, enabling them to evaluate how untreated, dyed, and bleached hair interact with smoke residues. The module emphasizes critical thinking through data interpretation, allowing students to draw informed forensic conclusions based on surface charge variations and chemical structure changes. Students also gain experience in experimental design, including sample preparation, controlled exposure, and proper use of analytical instrumentation, while reinforcing the importance of safety and scientific rigor. By connecting these techniques to actual forensic scenarios, such as custody disputes, workplace exposure investigations, and criminal cases, students enhance their ability to apply analytical methods in legal and investigative contexts. Additionally, the module fosters collaborative learning and self-reflection, with pre- and postlab surveys guiding students to assess their conceptual understanding, technical skills, and confidence in using forensic chemistry tools.

## Instructor and Lab Assistant Preparation Tasks

### Hair Sample Preparation

Healthy untreated hair samples were donated by a 23-year-old Hispanic female at University of Texas at El Paso. The hair was thoroughly washed with shampoo and allowed to air-dry naturally before any further treatments. A total of 1 g of hair was separated into three distinct groups: untreated (control), bleached, and dyed, with each group containing three samples (n = 3). The untreated hair served as the control group to establish a baseline for comparison. These hair samples underwent no additional chemical treatments, ensuring their natural state was preserved for subsequent analyses. Hair samples were stored securely in sealed glass containers at room temperature between weekly sessions.

### Bleaching Process

A commercial bleaching agent was utilized that included hydrogen peroxide (H_2_O_2_) and ammonium persulfate as its active components, known for their effective disintegration of natural pigments in hair. The preparation of the bleaching mixture was conducted in strict accordance with the manufacturer’s instructions to ensure accuracy and reproducibility. Once prepared, the 1 g of hair strands were soaked into bleach for 30 min to ensure the bleaching effect while maintaining the structural integrity of the hair. This consistency in timing across samples was critical to ensure that any subsequent differences in smoke deposition could be attributed to the hair’s condition rather than variability in the bleaching process. Following the bleaching, each sample was thoroughly rinsed with deionized (DI) water to remove the residual of bleaching agents, which could potentially affect the results. Finally, the samples were air-dried at room temperature for 48 h and prepared to be placed in controlled exposure to cigarette smoke.

### Dyeing Process

For the dyeing procedure, we selected a black hair dye known for its robust color retention and compatibility with various hair types. Each dye batch was prepared following the manufacturer’s instructions to ensure that the chemical properties were correctly aligned for optimal application. The dye was applied uniformly to each hair sample, a critical step to ensure that the coloration would be consistent across all samples, minimizing any variability that could impact the study’s outcomes. We allowed the dye to set on the hair for a precisely timed duration of 10 min as instructed by the supplier. Subsequently, treatments were placed in a sieve and rinsed thoroughly with DI water and shampoo. This rinsing continued until the runoff water was completely clear, to ensure the retention of dye residue on hair strands. Hair samples were air-dried at room temperature, similar to the previous stage before exposure to cigarette smoke.

### Hazards

The experimental procedure involved potential exposure to cigarette smoke, which contains harmful substances such as nicotine, tar, and carbon monoxide, all known respiratory irritants and carcinogens. Proper ventilation, fume hoods, and gas monitoring (e.g., BW CLIP 4 multigas detectors) were essential during controlled ETS exposure. Additionally, the use of methanol and hydrogen peroxide introduced flammable and oxidative chemical hazards, respectively. Strict adherence to PPE guidelines (gloves, goggles, lab coats) and chemical handling protocols was enforced throughout the experiment. Students were trained in handling these substances safely, and all procedures were supervised to minimize health and environmental risks.

### Demonstration Methods for Students (Three-Week Laboratory Activity)

#### Week 1 (3 Lab Sessions in 3 Days)

Students filled the prelab surveys to assess the learning outcomes of this demonstration before starting the experiments. In UV–visible spectroscopy analysis, students were introduced to the basic principles and operation of UV–visible spectroscopy. Next, students learned to carefully place hair strands in a glass Petri dish (soda-lime glass, H 12 mm, lid, diameter 40 mm) and then positioned within a polypropylene vacuum desiccator chamber manufactured by SP Scienceware. This experiment repeated for three hair types, day 1 virgin hair, day 2 dyed hair, and day 3 the bleached hair. To facilitate the passage of cables for an electrical burner and to allow oxygen diffusion, a hole was punctured in the chamber’s lid. Students weighed a subsample of cigarette tobacco (0.85 g) and spread it on a copper net (shown in Figure[Fig fig1]). This setup was exposed to a controlled smoking process at a temperature of 450 °C, administered by an electric burner across four distinct time intervals: 15, 30, 45, and 60 min. A control sample consisting of hair strands with no cigarette exposure was also maintained throughout the experiment for comparative analysis. Two glass bottles containing water and methanol were also placed in the chamber as controls to provide a comparative indicator of the smoke diffusion. The oxygen level and released CO were monitored in all experiments using the portable BW CLIP 4 multigas detector (Honeywell Inc.). The experimental set up is shown in Figure 1.

**Figure 1:**
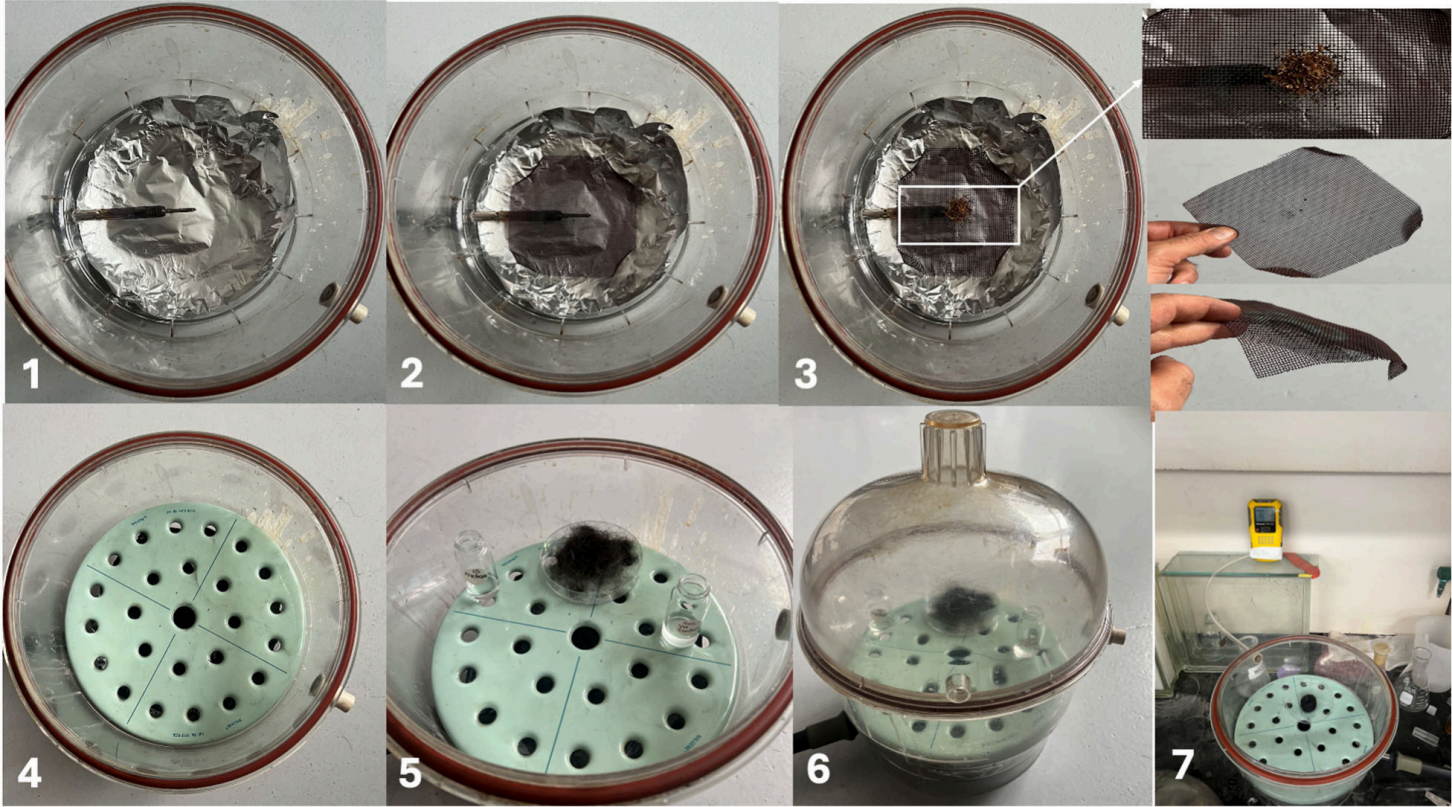
Experimental setup for hair exposure to cigarette smoke. (1) Preparation of the desiccator chamber with aluminum foil lining to prevent contamination. (2) Aluminum foil-lined chamber with an electrical burner placed at the center. (3) Placement of cigarette tobacco on a metal mesh above the burner. (4) Insertion of a perforated platform to hold hair samples above the smoke source. (5) Hair samples and control vials of water and methanol placed on the perforated platform. (6) Assembled desiccator chamber with a sealed lid to contain smoke during exposure. (7) Monitoring of oxygen levels and CO release using a portable BW CLIP 4 multigas detector during the smoking process.

Following each designated smoking interval, the experiment was paused to prepare the setup for the subsequent exposure period. Immediately after each exposure, the exposed hair strands were transferred to a glass tube and rinsed extensively with methanol. This process involved 2 min of vigorous shaking using a vortex mixer to ensure optimal dissolution of any tobacco smoke residues into the methanol. Postrinsing, the hair was carefully removed, and the methanol solution was preserved under refrigeration for subsequent UV–visible spectroscopy analysis. This methodology ensures a rigorous assessment of smoke residue adherence to hair, while the systematic cleaning and controlled experimental conditions aim to yield reliable, reproducible results for further analytical evaluation. Each student measured the absorption spectra of methanol solutions containing smoke residues extracted from untreated, dyed, and bleached hair samples exposed to ETS. Students will record and interpret the absorbance values at 280 nm to quantify nicotine and tar components.

### UV–Visible Spectroscopy Analysis

The concentration of smoke residues in the hair samples was quantified using UV–visible (UV–vis) spectroscopy (PerkinElmer) after extracting the adsorbed residues by rinsing the exposed hair samples with methanol three times. The resulting methanol extracts were scanned using a full wavelength range of 200–700 nm. Among the observed spectra, the absorbance at 280 nm was specifically monitored using second-order derivative processing.

The selection of 280 nm was based on its established association with strong π–π* transitions of aromatic compounds such as nicotine and polyaromatic hydrocarbons, which are prominent components of tobacco smoke. This wavelength consistently showed peak absorbance in our extracts across different hair types and exposure conditions, making it a reliable indicator for quantifying smoke residue deposition.

To ensure measurement accuracy, a baseline scan using deionized water was performed prior to sample analysis to subtract background noise. Spectra were collected at room temperature using a 2.5 nm sampling interval, fast scan speed, and 5.0 nm slit width. The UV–vis absorbance data at 280 nm were used to evaluate the extent of smoke residue adsorption on virgin, dyed, and bleached hair. Comparative analysis of these spectra provided insight into how cosmetic treatments influence the hair’s capacity to retain environmental smoke contaminants.

#### Week 2 (3 Lab Sessions in 3 Days)

In sample preparation and zeta potential analysis, students learned sample preparation techniques, including cutting, freezing, grinding, and suspending hair samples in DI water. Students learned the principle of dynamic light scattering (DLS). They used sonication and centrifugation methods to prepare hair suspensions. Zeta potential measurements conducted on untreated, dyed, and bleached hair samples before and after ETS exposure. This experiment repeated for three hair types, day 1 virgin hair, day 2 dyed hair and day 3 the bleached hair. Students interpreted surface charge variations associated with contaminant interactions.

### Zeta Potential Measurement

Hair samples were then cut into small pieces (<1 cm in length) using scissors to facilitate grinding. The cut hair pieces were placed into a mortar and a small amount of liquid nitrogen was added to freeze the hair, making it brittle and easier to grind. The pestle was used to grind the frozen hair into a fine powder until a uniform consistency was obtained. 0.1 g of the hair powder was weighed using an analytical balance and transferred to a clean 15 mL polypropylene tube. To prepare the suspension, we added 10 mL of deionized (DI) water to the tube containing the hair powder and sonicated the suspension for 10 min to ensure uniform dispersion. The suspension was then centrifuged at 3000 rpm for 5 min to remove any large aggregates, and the supernatant was collected for zeta potential measurement. The hair suspension supernatant was transferred to the sample cell of the analyzer. Zeta potential measurements (n = 3) were performed using Malvern Zetasizer (Malvern, UK). The data was analyzed to determine the average zeta potential of the hair powder suspension, and the values for untreated hair were compared with those for dyed and bleached hair.

#### Week 3 (3 Lab Sessions in 3 Days)

This week, students explored the principles and applications of Fourier Transform Infrared Spectroscopy (FTIR). During the lab sessions, they analyzed untreated, dyed, and bleached hair samples to assess chemical modifications resulting from exposure to ETS. Using FTIR, students identified changes in key spectral regions, specifically 3000–2800 cm^–1^ and 1700–1500 cm^–1^, which are indicative of alterations in hair structure and composition. The experiment was conducted over three consecutive days, each focusing on a different hair type, day 1: virgin hair, day 2: dyed hair and day 3: bleached hair. This hands-on series allowed students to practice spectral interpretation and deepen their understanding of how chemical treatments and environmental exposures affect biological samples.

### FTIR Analysis

The changes in functional groups of hair samples before and after smoke exposure were evaluated using FTIR at room temperature. The FTIR analysis adhered to the ASTM E1252 standard procedure,^29^ employing the Thermo Scientific Nicolet iS5 FT-IR Spectrometer.^30^ During the linear scan mode, the sampling depth varied across the spectrum. With an optical velocity of 0.47 cm/s, the Fourier frequency ranged from 470 to 940 Hz, covering a spectral range of 400 to 3000 cm^–1^.

#### Week 4

Feedback and evaluation 13 students from diverse majors (forensic science with concentrations in biology and chemistry, environmental science, and engineering) participated in an evaluation session to provide feedback on the effectiveness of the demonstration methods. Students completed pre- and postlab surveys to assess learning outcomes, practical skills gained, and suggestions for method integration into forensic chemistry laboratory curricula.

### Student Feedback Survey

A pre- and postlab survey was conducted with 13 volunteer student researchers from diverse disciplines, including environmental science, forensic biology, forensic chemistry, and chemistry and biochemistry, to assess their learning outcomes and engagement in forensic analytical techniques. The survey was part of an experiment on quantifying hair exposure to environmental tobacco smoke, conducted in the forensic lab at the University of Texas at El Paso. The prelab survey assessed baseline knowledge, confidence levels, and expectations, while the postlab survey measured improvements in understanding, technical skills, and application of UV–vis spectroscopy, FTIR analysis, and zeta potential measurement. Data was collected through hard copy printed questionnaires. It provided insights into student learning and the effectiveness of the developed forensic methodology. The student feedback questionnaire for pre- and post-Lab experiments, provided in section S4- Table S1, shows the structured questions used for assessment.

## Results and Discussion

All analytical results presented in this article are representative of one student group’s findings. However, each group of 2–3 students conducted their own experiments, obtained independent results, and performed interpretations as per the instructional guidelines.

### Inquiry-Based Scientific Comprehension of Week 1 Lab

This laboratory module provided students with an authentic, inquiry-based experience that integrated real-world forensic science with fundamental spectroscopy techniques. By analyzing how different hair treatments influence smoke residue retention, students developed a deeper conceptual understanding of chemical interactions and environmental exposure assessments. The hands-on use of UV–visible spectroscopy fostered both technical competence and data interpretation skills, while the experimental design encouraged students to critically assess variables such as hair treatment, exposure time, and matrix effects.

Moreover, this activity strengthened students’ ability to translate spectroscopic data into forensic conclusions, preparing them for professional scenarios such as custody disputes, workplace exposure investigations, or crime scene analysis. The clear differentiation in results across hair types allowed students to directly observe how physical and chemical modifications affect biological material behavior, a key concept in forensic chemistry and environmental toxicology. Overall, this lab enriched students’ scientific reasoning, promoted learning, and reinforced the value of interdisciplinary approaches in applied forensic education.

As shown in Figure 2, the absorbance values recorded at the wavelength of 280 nm indicated potential absorption of nicotine and tar components with a positive correlation to the time of exposure within a 60 min interval. Hair treated with permanent dye displayed the highest levels of smoke residue deposition across all exposure intervals. The UV–visible absorbance spectra revealed a significant increase in smoke residue absorption compared to virgin and bleached hair, by 1.8 and 3.2 times higher at 60 min, respectively.

**Figure 2:**
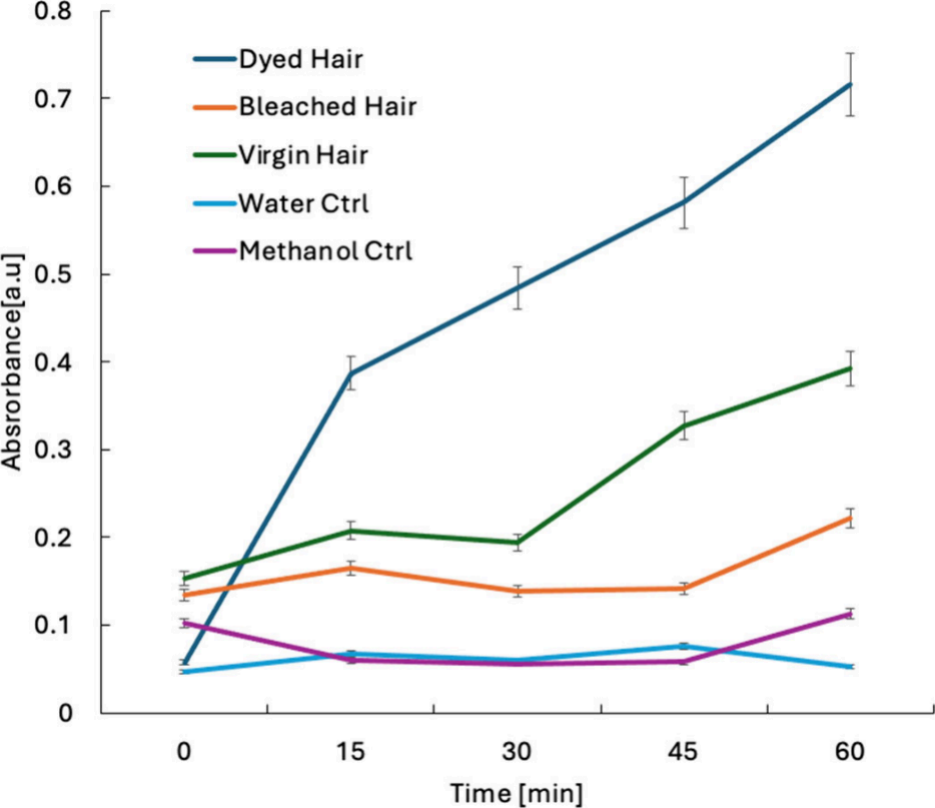
Absorbance of smoke residues dissolved in methanol from different hair types over time. The graph shows the absorbance 280 nm corresponding to nicotine and tar components on hair within 15, 30, 45, and 60 min.

The dyeing process, which alters the hair’s surface chemistry, may have introduced new functional groups or increased porosity, creating more binding sites for smoke particles and thus enhancing the retention of nicotine and tar-associated components. Virgin (untreated) hair exhibited a progressive increase in smoke residue absorption in proportion to exposure time, while bleached hair showed the lowest absorbance peaks, indicating that the bleaching process altered the hair’s physiochemical structure and reduced its ability to adsorb smoke particles.

A comparative analysis of the three hair types revealed a clear hierarchy of smoke residue affinity: dyed hair retained the most, followed by bleached hair, then virgin hair. This suggests that cosmetic chemical treatments significantly impact the adsorption properties of hair and must be accounted for in forensic exposure assessments. Control solutions of water and methanol placed in the desiccator chamber showed negligible contamination, supporting the integrity of the experimental setup. The initial (time zero) absorbance values exhibited clear differences among the untreated (virgin), dyed, and bleached hair samples, as demonstrated in Figure 2. These differences likely result from intrinsic chemical and structural alterations introduced by cosmetic treatments prior to tobacco smoke exposure. Specifically, dyed and bleached hair undergo oxidative and chemical modifications, affecting the hair’s surface porosity and residual organic composition. These changes facilitate the leaching of residual dyes, dye precursors, or aromatic protein degradation products into methanol during the initial rinsing step, even before exposure to tobacco smoke. In contrast, untreated hair retains its native structure and contains fewer extractable residues, resulting in a notably lower baseline absorbance. Furthermore, variations in light scattering due to surface roughness and structural porosity across the hair types may also contribute to the observed baseline spectral differences. Thus, absolute absorbance at time zero is not directly compared; instead, the focus remains on relative absorbance changes over the exposure period to reliably assess smoke residue adsorption across different hair types.

### Scientific Comprehension of Week 2 Lab

Figure 3 shows the impact of cigarette smoke on untreated, dyed, and bleached hair samples using FTIR spectroscopy. This technique allowed students to investigate molecular-level changes in hair composition after exposure to ETS, providing a hands-on opportunity to connect theory with application. Figure 3A presents FTIR spectra of untreated hair (black line) and smoke-exposed untreated hair (red line). After exposure, noticeable new peaks and shifts in transmittance intensities emerge, indicating chemical alterations. Changes were especially prominent in the 3000–2800 cm^–1^ region (C–H stretching of alkanes) and the 1700–1500 cm^–1^ range (Amide I and II bands), which reflect modifications to the protein backbone of hair. These spectral changes served as real-time evidence of environmental interaction with biological samples. Figure 3B shows similar comparisons for dyed hair, where exposure to smoke caused intensified shifts in the 3500–3200 cm^–1^ region (O–H and N–H stretching vibrations) and 1600–1500 cm^–1^ (Amide II region). These alterations suggest chemical interactions between dye constituents and smoke residues. The elevated response in dyed hair indicates a higher reactivity due to prior chemical treatment.

**Figure 3:**
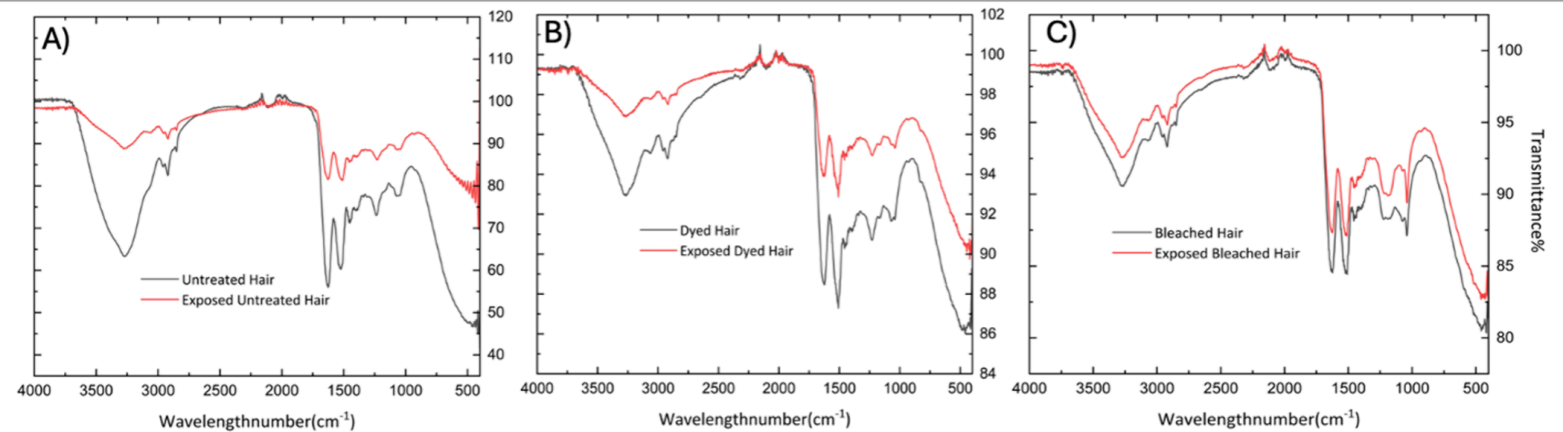
FTIR spectra of hair samples exposed to cigarette smoke. (A) Untreated hair compared to untreated hair exposed to cigarette smoke. (B) Dyed hair compared to dyed hair exposed to cigarette smoke. (C) Bleached hair compared to bleached hair exposed to cigarette smoke.

Figure 3C compares bleached hair before and after smoke exposure, with changes spanning 500–3200 cm^–1^. The broadening of peaks in the O–H stretching region and changes in Amide I and II bands point to structural degradation and protein damage induced by the dual effects of bleaching and smoke exposure. The increased porosity of bleached hair likely enhances its susceptibility to smoke residue adsorption. From an educational standpoint, this FTIR module reinforced critical analytical thinking by guiding students through spectral interpretation of real biological samples. Rather than working with abstract compounds, students explored how cosmetic alterations change a material’s chemical behavior and how forensic tools like FTIR can detect those changes. By comparing spectra across different hair conditions, students practiced identifying functional groups and recognizing patterns in complex data sets, skills directly transferrable to forensic, biomedical, and environmental chemistry fields.

Furthermore, this hands-on experience emphasized the interdisciplinary nature of forensic chemistry, combining biology, toxicology, and analytical instrumentation. Students gained confidence in interpreting real-world data and understanding how molecular-level changes relate to broader questions about environmental exposure and personal history, key themes in modern forensic investigations. This lab taught FTIR instrumentation also illustrated its power in addressing societal and legal questions through science.

### Scientific Comprehension of Week 3 Lab

This experiment enabled students to apply dynamic light scattering principles to a biological matrix, an approach not commonly encountered in early forensic training. By preparing suspensions, grinding hair, and interpreting electrophoretic mobility data, students bridged the gap between physical chemistry concepts and real-world forensic applications. Zeta potential analysis introduced students to an advanced yet accessible method of understanding how changes in surface charge affect interactions with contaminants. This is a foundational concept in environmental toxicology, biophysics, and forensic trace analysis. Students learned to connect numeric values with physical phenomena, reinforcing the concept that forensic evidence is not just about presence or absence, but about surface interactions and material behavior.

The zeta potential measurements for untreated, dyed, and bleached hair samples both in control and smoke-exposed conditions are illustrated in Figure 4. Zeta potential serves as a critical indicator of hair surface charge, helping reveal how chemical treatments and smoke exposure alter hair’s electrostatic properties. This analysis introduced students to the importance of surface chemistry in forensic science and environmental exposure studies. Figure 4A shows that untreated hair under control conditions exhibits a slightly positive zeta potential of 1.7 mV. After exposure to cigarette smoke, the value drops significantly to −6.2 mV, suggesting the adsorption of negatively charged smoke residues. This shift demonstrates how untreated hair interacts with environmental pollutants and served as a baseline for comparison. Figure 4B highlights dyed hair, which starts with a zeta potential of 0.4 mV, lower than untreated hair, likely due to changes in surface chemistry caused by dyeing. Postexposure, the zeta potential increases to 2.6 mV, reflecting an unusual positive shift. This suggests the presence of positively charged functional groups from dye molecules that attract or interact differently with smoke residues. Students were encouraged to think critically about how cosmetic treatments may alter the surface charge landscape of biological samples and lead to varying forensic interpretations.

**Figure 4:**
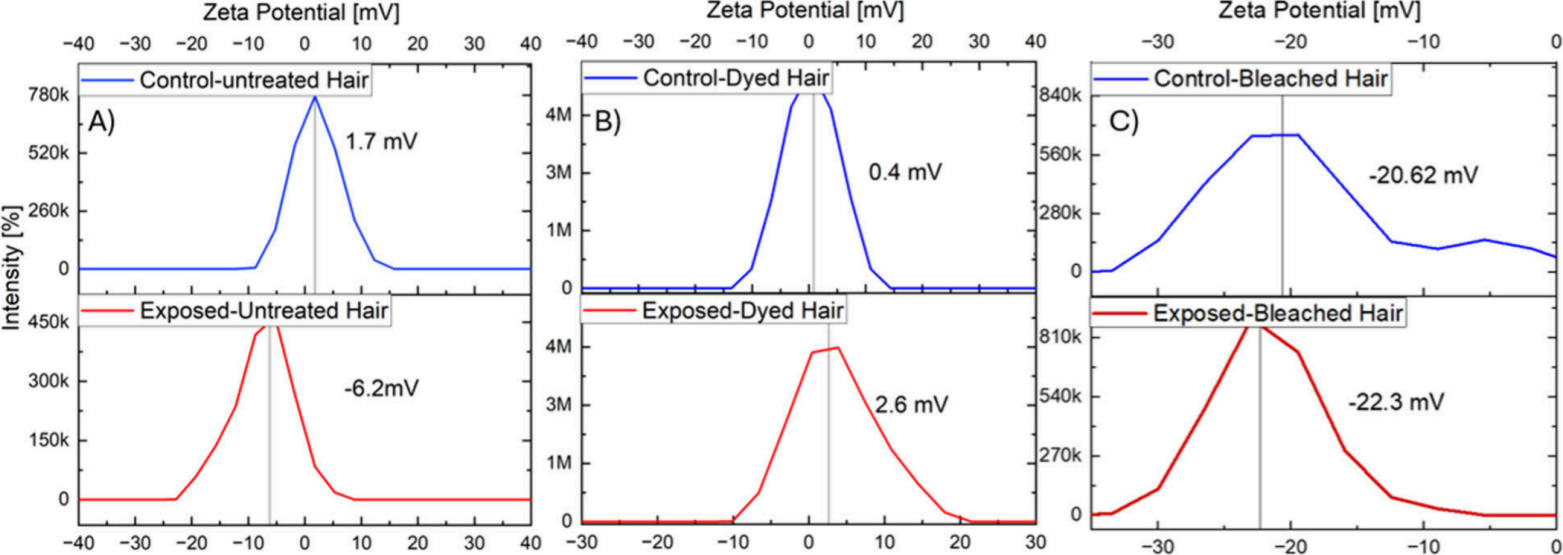
Zeta potential measurements of hair samples before and after smoke exposure for untreated, dyed, and bleached hair samples in control (blue) and smoke-exposed (red) conditions.

Figure 4C reveals that bleached hair exhibits a highly negative zeta potential of −20.62 mV even before exposure, reflecting its high porosity and structural disruption. Upon smoke exposure, the value becomes even more negative at −22.3 mV, indicating strong affinity for negatively charged smoke particles. Students observed that bleaching not only affects cosmetic appearance but also significantly enhances the surface’s reactivity, making bleached hair more prone to retaining environmental contaminants. In summary, the lab not only demonstrated a clear hierarchy of smoke residue affinity of bleached > dyed > untreated but also fostered a deeper understanding of how analytical chemistry techniques illuminate biological and environmental interactions. It strengthened students’ abilities to hypothesize, interpret, and reason through the multidimensional behavior of evidence, a key skill in modern forensic science.

### Student Outcomes

In this project, 13 volunteer students successfully demonstrated the laboratory method for quantifying hair exposure to ETS using forensic analytical techniques. The prelab and postlab assessments (shown in Table S1 and Figure 5) provided insights into the effectiveness of this experimental demonstration as an educational tool for forensic chemistry. Two student lab report examples are provided in Supporting Information (SI), shown in sections S2: Example Student Report 1 and S3: Example Student Report 2.

**Figure 5:**
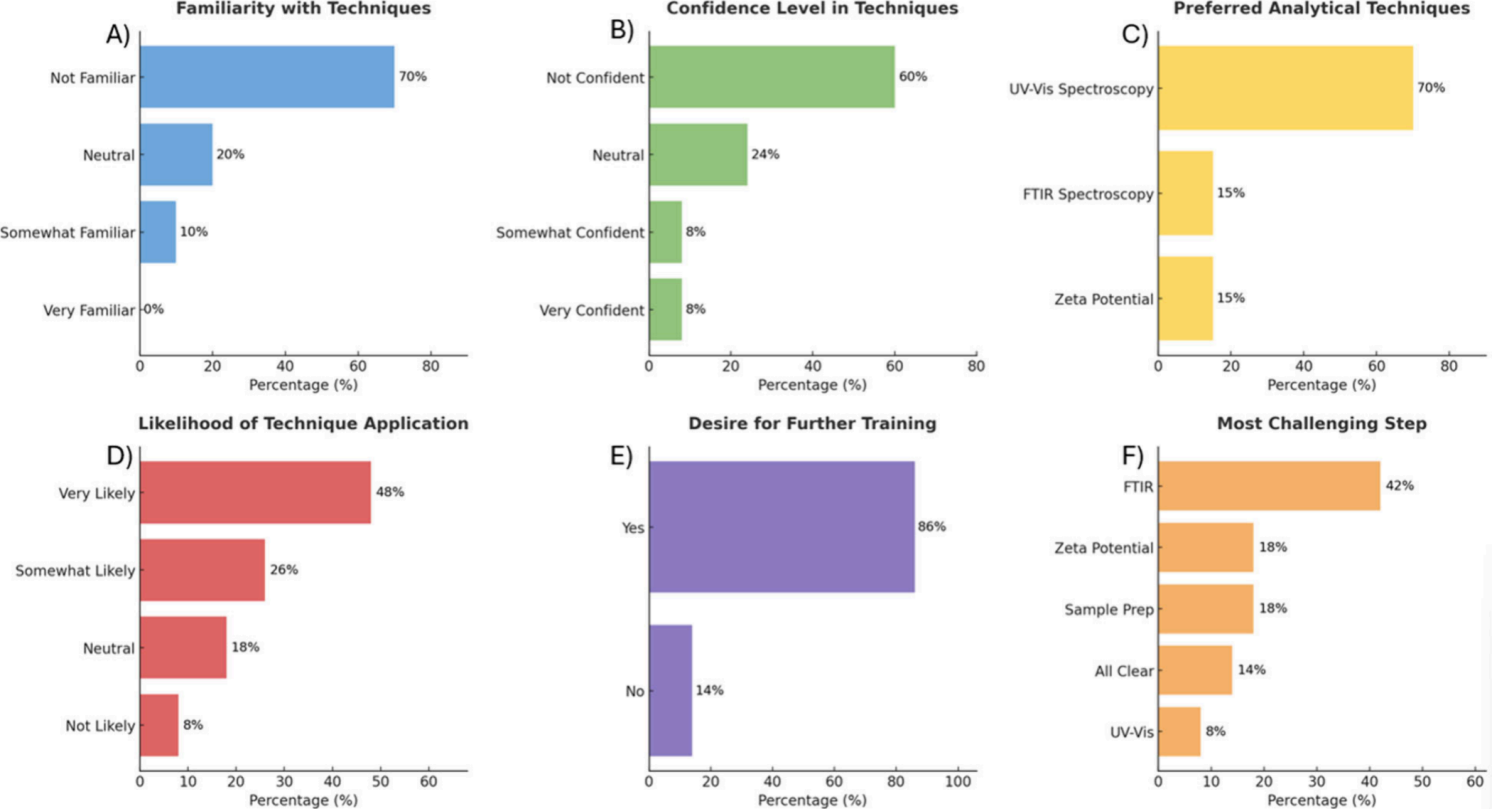
Student responses to prelab and postlab assessments evaluating the effectiveness of the forensic hair analysis module. (A) Prelab familiarity with forensic hair analysis. (B) Prelab confidence in analyzing chemical contaminants in hair samples. (C) Postlab identification of the most effective analytical technique for detecting smoke residues. (D) Postlab likelihood of students using the learned forensic techniques in future applications. (E) Postlab student confidence in desire for further training. (F) Postlab identification of the most challenging analytical technique experienced by students.

Before conducting the experiment, as shown in Figure 5A a majority of the students (70%) reported minimal familiarity with forensic hair analysis, with only 10% expressing some familiarity. Also confidence level analysis in Figure 5B showed majority are not confident in using analytical techniques. Students initially recognized hair analysis predominantly for identifying drug-use history (60%) but underestimated its utility for environmental exposure assessments (15%). Most students correctly identified Gas Chromatography–Mass Spectrometry (GC-MS) as a common analytical technique (64%), while fewer acknowledged the relevance of UV–visible and FTIR spectroscopy.

Postlab assessment demonstrated a marked improvement in understanding. Approximately 84% of students reported significantly improved familiarity with forensic hair analysis. As shown in Figure 5C, the UV–visible spectroscopy was identified by 70% of students as the most effective technique for detecting smoke residues, reinforcing the effectiveness of spectroscopic methods in forensic environmental assessments. All students identified dyed hair as having the highest deposition of smoke residues, confirming a clear understanding of how chemical treatments impact residue adsorption. Zeta potential analysis (Table S1) was particularly enlightening, with 43% of students correctly identifying its primary use as measuring changes in surface charge due to contaminant interaction. The hands-on experience with FTIR spectroscopy, though found challenging by 42% of students (Figure 5F), substantially enhanced their analytical skills. However, student inquiry was evident through divergent interpretations of spectral data and smoke residue patterns, some groups emphasized chemical affinity to melanin, while others focused on hair surface morphology. These varied conclusions, drawn with minimal instructor intervention, demonstrate that students moved beyond procedural tasks into hypothesis generation and critical reasoning.

The experimental design effectively allowed all students to compare results across different hair treatments. The hands-on exposure provided clarity in understanding the interactions between ETS residues and chemically treated hair. Students noted the most valuable component was learning how chemical modifications like dyeing and bleaching alter the hair’s physical and chemical properties, impacting forensic interpretations. Postlab analysis shown in Figure 5D,E further revealed the likelihood of students using the learned forensic techniques in future applications was nearly 50% with majority stating the need for advanced training.

Students recommended including more diverse chemical treatments and repeated exposure scenarios to enhance future experiments’ educational value. Overall, this demonstration significantly increased students’ practical and theoretical knowledge, underscoring its potential application in real forensic investigations such as custody cases, workplace exposure assessments, and criminal investigations involving environmental tobacco smoke.^31^

## Conclusion

This work delivers a practical, noninvasive laboratory module that quantifies hair exposure to environmental tobacco smoke while building core forensic-analytical competencies. Using controlled smoke exposure coupled with UV–visible spectroscopy, FTIR, and zeta potential measurements, students were able to detect and interpret chemical signatures of ETS on untreated, dyed, and bleached hair. Across techniques, cosmetic treatment was a decisive factor: treated hair exhibited distinct surface chemistry and structural changes that altered residue interactions and detectability. In our pilot, dyed hair frequently showed higher methanol-extractable residues by UV–Vis, whereas bleaching produced marked shifts in functional groups and more negative surface charge, indicating treatment-dependent mechanisms of adsorption and retention. These complementary observations reinforce the evidentiary value of multimodal analysis in exposure assessment and demonstrate how matrix history (cosmetic treatment) must be considered in forensic interpretation.

Pedagogically, the module achieved measurable learning gains. Pre/post assessments showed increased familiarity with forensic hair analysis, improved confidence with instrumental methods, and stronger capacity to translate spectra and surface-charge data into defensible conclusions. Because the workflow uses accessible instrumentation, clearly defined safety practices, and inexpensive materials, it is readily scalable for undergraduate teaching laboratories and adaptable to varied forensic scenarios (e.g., custody disputes, workplace exposure, scene reconstruction). Future iterations should incorporate quantitative calibration for specific ETS markers (e.g., nicotine/cotinine), broaden donor hair diversity and treatment histories, examine repeated/longer exposures and alternative smokes (e.g., cannabis), and evaluate decontamination protocols. Method validation across cohorts and institutions, coupled with structured interpretation frameworks, will further strengthen reliability and courtroom utility. Overall, the module integrates authentic forensic inquiry with robust analytics, providing a transferable teaching and investigative tool for assessing individual ETS exposure in hair.

## Supplementary Material





## Notes

S1 – Student’s handout for five-week lab assignments, providing detailed instructions, weekly objectives, materials, and procedures for conducting the ETS–hair exposure laboratory experiment using UV–vis, FTIR, and zeta potential analysis; S2 – Example Student Report 1, containing a complete sample lab report demonstrating data interpretation, UV–vis and FTIR results, zeta potential findings, and discussion of ETS adsorption differences across untreated, dyed, and bleached hair; S3 – Example Student Report 2, providing an additional full student-style report emphasizing forensic implications, analytical technique comparison, and interpretation of ETS residue interactions with cosmetically treated hair; S4 – Student feedback questionnaire for pre- and postlab experiments; and Table S1, the survey instrument used to assess baseline knowledge, skills gained, confidence levels, and perceptions of forensic hair analysis before and after the experiment (PDF, DOCX)
