# Supplementary material for: Developing and Demonstrating a Lab Method for Quantifying Hair Exposure to Environmental Tobacco Smoke with a Forensic Perspective
Source: J Chem Educ. 2025 Dec 25;103(1):479–87. doi: 10.1021/acs.jchemed.5c00479 (PMC12805574; doi:10.1021/acs.jchemed.5c00479)
Supplement: Supplementary file 2 [file ed5c00479_si_002.docx]

**Supplementary Information**

**Developing and Demonstrating a Lab Method for Quantifying Hair Exposure to Environmental Tobacco Smoke with Forensic Perspective**

Tanique Z Jones^1,2^, Christopher J Trejo^1,2^, Somayeh Mohammadi^1^, Hamidreza Sharifan^1,2,3^*

^1,2^Department of Chemistry and Biochemistry, University of Texas at El Paso, El Paso, Texas 79968, United States

^2^Forensic Science Program, University of Texas at El Paso, El Paso, Texas 79968, United States^3^Environmental Science and Engineering Program, University of Texas at El Paso, El Paso, Texas 79968, United States

*hsharifan@utep.edu

**List of content:**

S1-Student’s handout for five weeks

S2-Example Student Report 1

S3-Example Student Report 2

S4-Student feedback questionnaire for pre- and post-Lab experiments

**S1-Student’s handout**

**Course:** Forensic Chemistry /Environmental Science
**Objective:**
This laboratory assignment aims to introduce students to forensic analytical techniques for assessing environmental tobacco smoke (ETS) exposure using hair samples. Students will analyze untreated, dyed, and bleached hair exposed to ETS and evaluate the deposition of smoke residues using UV-visible spectroscopy, Fourier Transform Infrared Spectroscopy (FTIR), and Zeta Potential Analysis.

**Week 1: Hair Sample Preparation and Controlled Exposure to Cigarette Smoke**

**Objective:** Prepare hair samples and expose them to controlled ETS conditions.

**Materials:**

- - Virgin, dyed, and bleached human hair samples
  - Commercial hair bleach and black dye
  - Cigarette tobacco (measured 0.85 g per exposure)
  - Desiccator chamber with perforated platform
  - Electric burner (set at 450°C)
  - Glass vials containing DI water and methanol (for control)
  - Portable multi-gas detector (for CO and O₂ monitoring)

**Procedure:**

- 1. Separate hair samples into three groups: untreated, dyed, and bleached.
  2. Place samples in the controlled smoking chamber.
  3. Expose the hair to cigarette smoke at different time intervals (15, 30, 45, and 60 minutes).
  4. Collect and store hair samples for chemical analysis in subsequent weeks.
  5. Record initial observations on hair discoloration, texture changes, and odor retention.

**Week 2: UV-Visible Spectroscopy Analysis**

**Objective:** Quantify smoke residue deposition on hair samples.

**Materials:**

- - UV-Vis Spectrophotometer (PerkinElmer)
  - Methanol solution (for extracting smoke residue)
  - Glass cuvettes
  - Vortex mixer and centrifuge

**Procedure:**

- 1. Cut 0.2 g of each exposed hair sample into small pieces.
  2. Soak each sample in 5 mL methanol and vortex for 2 minutes.
  3. Centrifuge at 3000 rpm for 5 minutes and transfer the supernatant.
  4. Record absorbance spectra at 280 nm, corresponding to nicotine and tar components.
  5. Compare absorbance values for untreated, dyed, and bleached hair.
  6. Plot absorbance vs. exposure time to analyze trends.

**Week 3: FTIR Spectroscopy Analysis**

**Objective:** Identify chemical modifications in hair samples due to smoke exposure.

**Materials:**

- - FTIR Spectrometer (Thermo Scientific™ Nicolet iS5)
  - ATR (Attenuated Total Reflectance) module

**Procedure:**

- 1. Dry hair samples completely before FTIR analysis.
  2. Scan unexposed and exposed hair samples across 4000–400 cm⁻¹ spectral range.
  3. Identify changes in peak intensities, particularly in amide I (1700 cm⁻¹) and amide II (1500 cm⁻¹) regions.
  4. Compare spectra between hair types to evaluate smoke residue interactions.

**Week 4: Zeta Potential Measurements and Final Report**

**Objective:** Determine surface charge variations due to smoke exposure.

**Materials:**

- - Malvern Zetasizer for Zeta Potential Analysis
  - Liquid nitrogen, mortar, and pestle
  - DI water

**Procedure:**

- 1. Freeze and grind hair samples into a fine powder.
  2. Suspend 0.1 g of hair powder in 10 mL DI water and sonicate for 10 minutes.
  3. Centrifuge at 3000 rpm for 5 minutes, then analyze the supernatant for zeta potential.
  4. Compare surface charge variations before and after exposure.

**Week 5: Data Analysis and Final Report (Submission)**

Each student will submit a report that includes:

1. **Introduction:** Importance of ETS exposure assessment using hair.
2. **Methods:** Summary of experimental procedures.
3. **Results:** Graphs and tables from UV-Vis, FTIR, and zeta potential measurements.
4. **Discussion:**
   - Compare adsorption trends in untreated vs. chemically treated hair.
   - Explain how hair porosity, dye molecules, and smoke residues interact.
   - Discuss real-world forensic applications of the method.
5. **Conclusion:** Key findings and potential improvements for forensic investigations.

**Instructor Notes:**

- Ensure students work in ventilated lab conditions when handling cigarette smoke.
- Encourage students to discuss forensic implications in real cases.
- Promote teamwork for data interpretation and result validation.

**S2-Example Student Report 1**

**Title:** Comparative Analysis of Environmental Tobacco Smoke Adsorption on Untreated, Dyed, and Bleached Hair Samples

**Introduction**
Hair is an established forensic matrix for reconstructing long-term exposures due to its ability to adsorb and retain contaminants. This study explored how untreated, dyed, and bleached hair interact with environmental tobacco smoke (ETS), using UV–Vis, FTIR, and zeta potential analysis. The goal was to assess how chemical treatments influence residue deposition and forensic interpretation.

**Methods**

- **Exposure:** Hair samples (0.2 g each) exposed to ETS for 15–60 min.
- **UV–Vis:** Methanol extracts analyzed at 280 nm.
- **FTIR:** ATR-FTIR scans from 4000–400 cm⁻¹, focusing on amide I/II.
- **Zeta Potential:** Powdered hair suspended in DI water, sonicated, and analyzed.

**Results**

- **UV–Vis:** Absorbance increased with exposure time (Figure 1). Dyed hair consistently showed the highest absorbance (~0.42 at 60 min) vs. untreated (~0.25) and bleached (~0.12).
- **FTIR:** ETS exposure increased carbonyl peak intensity (1700 cm⁻¹) in dyed hair; bleached hair spectra showed reduced amide II intensity, suggesting structural degradation.
- **Zeta Potential:** Untreated hair shifted from −25 mV to −18 mV post-exposure; dyed hair shifted from −22 mV to −10 mV, indicating higher surface interactions.

**Discussion**
Dyed hair adsorbed the most ETS residues due to increased porosity and possible chemical binding between dye molecules and smoke components. Bleaching decreased adsorption capacity, likely due to cuticle damage reducing binding sites. FTIR confirmed structural alterations, while zeta potential highlighted changes in surface charge.

**Conclusion**
This experiment demonstrated the influence of hair treatments on ETS residue retention. Dyed hair poses challenges in forensic interpretation since it disproportionately retains smoke residues, which may exaggerate exposure estimates.

**S3-Example Student Report 2**

**Title:** Forensic Applications of Hair Analysis in Environmental Tobacco Smoke Exposure

**Introduction**
ETS exposure is linked to adverse health outcomes and can be detected in biological matrices. Unlike blood or urine, hair provides a long-term record, making it valuable for forensic casework. This report evaluates ETS adsorption on different hair types using three complementary analytical techniques.

**Methods**
Hair samples (virgin, dyed, bleached) were exposed to controlled ETS for up to 60 min. Methanol extracts were tested by UV–Vis, samples were analyzed by FTIR, and ground suspensions were analyzed for zeta potential.

**Results**

- **UV–Vis:** Peak absorbance at 280 nm correlated with exposure duration. Dyed hair showed the steepest slope (R² = 0.96).
- **FTIR:** Amide I/II peaks decreased in bleached hair after exposure, while dyed hair exhibited additional aromatic bands consistent with smoke residues.
- **Zeta Potential:** Post-exposure, dyed hair shifted from −20 mV to −8 mV, showing significant reduction in negative surface charge.

**Discussion**
These findings reinforce the importance of considering cosmetic treatments in forensic investigations. Dyed hair retained more ETS due to combined effects of porosity and molecular affinity. FTIR provided molecular evidence of nicotine/tar incorporation. Zeta potential offered surface-level confirmation of charge alteration by contaminants.

**Conclusion**
This lab demonstrated how forensic chemistry techniques can be applied to environmental exposure assessment. Results confirmed that dyed hair retains more ETS than untreated or bleached hair, underscoring the need for careful interpretation in casework.

**S4-****Student feedback questionnaire of pre- and post-Lab experiments**

Table S1. Pre and Post Lab assessment survey.

| **Pre-Lab Assessment (Before the Experiment)** | Responses [%] |
| --- | --- |
| **Section 1: Baseline Knowledge** | |
| 1.1 How familiar are you with forensic hair analysis before this experiment? | |
| Very Familiar | 0 |
| Somewhat Familiar | 10 |
| Neutral | 20 |
| Not Familiar | 70 |
| 1.2 What forensic applications do you think hair analysis is most useful for? *(Select all that apply)* | |
| Identifying drug use history | 60 |
| Determining environmental exposures | 15 |
| Confirming dietary intake | 10 |
| Establishing DNA profiles | 5 |
| 1.3 Which chemical techniques are commonly used for analyzing contaminants in hair samples? *(Select all that apply)* | |
| UV-Visible Spectroscopy | 8 |
| FTIR Spectroscopy | 12 |
| Gas Chromatography-Mass Spectrometry (GC-MS) | 64 |
| X-ray Diffraction (XRD) | 16 |
| 1.4 Which of the following factors can affect the deposition of environmental contaminants on hair? *(Select all that apply)* | |
| Hair type and porosity | 22 |
| Chemical treatments (dyeing, bleaching) | 18 |
| Length of exposure to contaminants | 36 |
| Washing hair frequently | 24 |
| 1.5 What is the main advantage of using hair analysis instead of blood or urine for detecting long-term exposure to toxins? | |
| Hair provides a permanent record of exposure | 16 |
| Hair analysis is faster than blood tests | 26 |
| Hair analysis detects only recent exposure | 17 |
| Blood and urine analysis are more reliable than hair analysis | 41 |
| **Section 2: Expectations and Confidence Levels** | |
| 2.1 What do you expect to learn from this experiment? *(Short answer)* |  |
| 2.2 How confident are you in your ability to analyze hair samples for chemical contaminants? | |
| Very Confident | 8 |
| Somewhat Confident | 8 |
| Neutral | 24 |
| Not Confident | 60 |
| 2.3 What challenges do you anticipate in this forensic analysis? *(Short answer)* |  |
| **Post-Lab Assessment (After the Experiment)** | |
| **Section 1: Knowledge Gained** | |
| 1.1 How has your familiarity with forensic hair analysis changed after completing this experiment? | |
| Significantly Improved | 84 |
| Moderately Improved | 16 |
| No Change | nr |
| Still Confused | nr |
| 1.2 What was the most valuable skill or concept you learned from this experiment? *(Short answer)* |  |
| 1.3 Which chemical technique did you find most effective for detecting smoke residues on hair? | |
| UV-Vis Spectroscopy | 70 |
| FTIR Spectroscopy | 15 |
| Zeta Potential Measurement | 15 |
| 1.4 How do different hair treatments (bleached, dyed, untreated) affect the adsorption of environmental contaminants? | |
| Dyed hair retains the most contaminants. | 100 |
| Bleached hair retains the most contaminants. | nr |
| Untreated hair retains the most contaminants. | nr |
| There was no significant difference. | nr |
| 1.5 How does zeta potential analysis help in understanding how contaminants interact with hair? | |
| It measures changes in surface charge of hair before and after exposure | 43 |
| It determines the amount of contaminants absorbed in hair | 32 |
| It detects DNA sequences in hair | nr |
| It quantifies the presence of chemical dyes in hair | 25 |
| **Section 2: Application and Reflection** | |
| 2.1 How likely are you to use the techniques learned in this experiment in future forensic applications? | |
| Very Likely | 48 |
| Somewhat Likely | 26 |
| Neutral | 18 |
| Not Likely | 8 |
| 2.2 Do you feel more confident in analyzing environmental contaminants using forensic chemistry techniques? | |
| Yes | 86 |
| No | 14 |
| **Section 3: Knowledge Assessment** |  |
| 3.1 What is the primary advantage of using hair analysis for environmental tobacco smoke (ETS) exposure assessment? | |
| a) Provides a real-time measurement of ETS exposure | 31 |
| b) Allows for long-term exposure assessment | 26 |
| c) Can detect exposure only within a few hours | 24 |
| d) Is less reliable than blood or urine analysis | 19 |
| 3.2 Which hair treatment showed the highest deposition of tobacco smoke residues in this experiment? | |
| a) Virgin (untreated) hair | nr |
| b) Dyed hair | 100 |
| c) Bleached hair | nr |
| d) All treatments showed equal deposition | nr |
| 3.3 What analytical technique was used to quantify tobacco smoke residues in this study? (Yes or No) | |
| a) Gas Chromatography (GC) | No |
| b) Fourier Transform Infrared Spectroscopy (FTIR) | Yes |
| c) UV-Visible Spectroscopy (UV-Vis) | Yes |
| d) X-ray Diffraction (XRD) | No |
| 3.4 How does bleaching affect the adsorption of ETS residues on hair? | |
| a) Increases deposition due to structural damage | nr |
| b) Decreases deposition by sealing the hair cuticle | 100 |
| c) Has no effect on smoke residue retention | nr |
| d) Neutralizes nicotine residues completely | nr |
| 3.5 Which factor primarily contributes to higher adsorption of ETS components in dyed hair? | |
| a) Increased porosity | nr |
| b) Chemical interaction of dye molecules with smoke residues | nr |
| c) Both a and b | 100 |
| d) None of the above | nr |
| **True or False Questions** | |
| 1. Hair analysis is a more reliable method than blood tests for determining long-term exposure to environmental tobacco smoke. (True / False) |  |
| 2. The UV-Visible spectroscopy technique was used in this experiment to analyze the chemical structure of nicotine and tar residues. (True / False) | 64% True  36% False |
| 3. Zeta potential analysis was used to measure the surface charge changes in hair samples before and after smoke exposure. (True / False) | 100% True |
| 4. The exposure time of hair to cigarette smoke had no significant effect on the amount of residue deposited. (True / False) | 100% False |
| 5. The experimental setup included a controlled chamber to simulate real-world ETS exposure in forensic applications. (True / False) | 52% True  48% False |
| **Section 4: Lab Experience & Practical Skills** | |
| 4.1 How confident are you in your understanding of how ETS residues interact with different hair types? | |
| Very Confident | 56 |
| Somewhat Confident | 26 |
| Neutral | 18 |
| Not Confident | nr |
| 4.2 How effectively did this experiment enhance your understanding of forensic analytical techniques (e.g., UV-Vis, FTIR, zeta potential analysis)? | |
| Extremely Effective | 52 |
| Moderately Effective | 18 |
| Neutral | 18 |
| Slightly Effective | 12 |
| Not Effective | nr |
| 4.3 Which technique did you find most challenging to understand? | |
| UV-Vis Spectroscopy | 8 |
| FTIR Spectroscopy | 42 |
| Zeta Potential Analysis | 18 |
| Sample Preparation and Exposure Methods | 18 |
| None – All Were Clear | 14 |
| 4.4 Did the experimental design allow you to compare results effectively across different hair treatments? | |
| Yes | 100 |
| No (Please Explain) | nr |
| **Section 5: Open-Ended Feedback** | |
| 5.1 What was the most valuable part of this experiment in helping you understand forensic applications of hair analysis? |  |
| 5.2 What improvements could be made to enhance the clarity and educational value of this lab? |  |
| 5.3 Do you have any suggestions for additional forensic experiments that could complement this study? |  |
| 5.4 How do you see this method being applied in real forensic cases? |  |
| 5.5 Any additional comments or feedback? |  |
